# Characteristics of the Spatial Location of Sports Facilities in the Northern Great Plain Region of Hungary

**DOI:** 10.3390/sports10100157

**Published:** 2022-10-18

**Authors:** Gábor Kozma, Károly Teperics, Klára Czimre, Zsolt Radics

**Affiliations:** Department of Social Geography and Regional Development Planning, University of Debrecen, H-4025 Debrecen, Hungary

**Keywords:** sports facilities, Northern Great Plain region settlements, spatial characteristics

## Abstract

Sports facilities play a very important role in educating people about the benefits of a healthy lifestyle, and the examination of their spatial distribution is one of the important research areas of sport geography, a field of study becoming increasingly important in recent times. In this spirit, the aim of this paper is to present the spatial distribution of sports facilities in a specific Hungarian sample area, the Észak-Alföld (Northern Great Plain) region, to point out the differences between settlements, as well as the reasons behind these differences. Data received from the local authorities and state administration bodies were used for the preparation of the study, which included the different sports facilities at the settlement level in addition to information found on the Internet. The following conclusions were drawn based on the research. First of all, it was found that the settlement size significantly influences the spatial distribution of sports facilities, inter alia, larger settlements with larger populations boast increased demand and higher purchasing power and also have more enhanced and more diverse sports infrastructure. Secondly, in the case of competitive sports, the size of settlements is less relevant; there are only insignificant differences between the settlements of different sizes. This can be explained by the fact that almost all settlements have their own football pitch. Thirdly, the administrative role of the settlements was also found to be significant since settlements being on higher levels of the hierarchy (district centres, county seats) always have better facilities.

## 1. Introduction

Providing adequate sporting opportunities for people is an increasingly important part of society for a number of reasons. On the one hand, it can create the conditions for a healthy lifestyle [1,2,3,4,5], which is also a very important factor for central governments in reducing health spending. On the other hand, sports facilities also create a community space [6,7,8,9,10,11] that brings members of society closer together, thus increasing cohesion between them.

However, the existence and easy accessibility of sports facilities of adequate quality is a prerequisite for achieving the above objectives [12,13,14,15,16]. In this spirit, it is not surprising that one of the important research areas of sport geography, which has gained increasing importance over the last 30 years [17,18,19], is the study of the spatial location of sports facilities and the analysis of access to them. Studies on the geographical distribution of sports facilities have fundamentally focused on two spatial levels: on the one hand, the researchers investigated the regularities of the location of facilities within settlements [20,21,22]; on the other hand, the differences between municipalities were analysed [23,24].

Among the studies analysing the location of sports facilities in cities/agglomerations, research in the United States of America [25,26] distinguished three types of location (city centre/downtown, within the city, edge of the city/suburbs), and explored the specificities of their evolution over time (for example, the recent rise of inner-city facilities). According to the study examining spatial development of sports facilities in Hungarian cities of county rank [27], larger sports facilities were especially constructed on the edge of cities or in the suburbs, while in the case of smaller facilities a bigger role was played by locations within the city boundaries. Studies carried out in Hamburg, Germany [28], underlined the importance of the income conditions characteristic of the individual neighbourhoods. The importance of income conditions was also highlighted by Billaudeau and his colleagues [29] as a result of their research in the Paris region, and they emphasised the differences between different types of sports facilities as well. Analysis on differences in the spatial location of sports facilities and population distribution in Nanning City (China) also highlighted the differences between different types of facilities [20]: basketball and table tennis courts were both equally well distributed, while fitness centres and swimming pools were the most unequally distributed sports facilities.

The analyses concerning the differences between the settlements yielded quite varied results. According to the findings of a survey of the situation in Scandinavian countries (Denmark, Norway), rural areas have better results in relative terms than urban areas [30]; at the same time, in the case of facilities appearing in the 1990s (e.g., fitness centres, ice rinks, multifunctional activity centres), which have made different, newer forms of physical activity possible, the advantage of cities and more densely-populated areas is quite clear. A research project exploring the conditions in the Netherlands yielded partly similar and partly different results [31]. On the one hand, it was also clearly shown that with the decreasing level of urbanization, the relative value (the number of facilities per 10,000 inhabitants) of the sports facilities was increasing; on the other hand, in terms of how widespread certain types of sports facilities (e.g., fitness centre, golf course, swimming pool, sport hall) are, no major differences could be established between the rural and the urban areas.

Analyses carried out by Higgs and his colleagues [32] in Wales highlighted the importance of the population’s deprivation levels: the areas most affected by deprivation are dominated mainly by public-owned sports facilities. Research concerning spatial distribution of constructed sports facilities [33] has indicated that the size of settlements can have a significant influence on the football fields completed, and the economic power of the local government has a major impact on their willingness to construct football fields in the first place. A Canadian research project [34] based on analyses on the level of settlements pointed out that the relatively higher value of large cities is followed by a lower value in the first suburban zone, while more distant suburbs once again have a higher value. 

**Figure 1:**
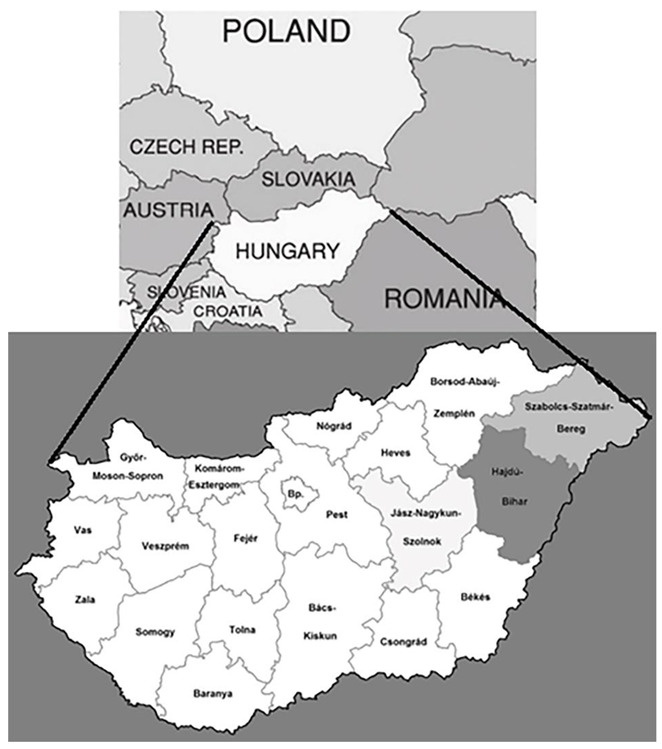
Geographical location of Northern Great Plain region and its counties. Source: own work.

The aim of our study, which relates to the latter research area, is to analyse the spatial location of sports facilities in a specific spatial unit, the North Great Plain region (Figure 1), which is located in Hungary and includes three counties, and to explore the factors influencing it.

## 2. Materials and Methods

The research was composed of several phases. In the first phase the range of settlements included in the study were selected. As a result, 96 settlements were selected from the 389 settlements of the region, while seeking to provide a sample fully representing the entire settlement structure from both an administrative and a population point of view (Hajdú-Bihar county—28 settlements; Jász-Nagykun-Szolnok county—28 settlements; Szabolcs-Szatmár-Bereg county—40 settlements). In the second phase, the list of sports facilities operated in the selected settlements was compiled using a wide range of methods—regarding that unfortunate fact that no such complex and detailed database currently exists in Hungary. Therefore, first, we used data supplied by the local governments, which mainly covered information on public sports facilities (e.g., swimming pools, sports halls). Secondly, we relied on the data provided by the regional school district centres which summarised the qualitative characteristics of the sports-related rooms in the schools (e.g., the size of gyms). Thirdly, the Internet was an important source of information, with the help of which—primarily by using data on privately-owned sports facilities (e.g., tennis courts, fitness centres)—we supplemented the above lists. In the end, 1109 sports facilities were included in the database.

During the analysis, different types of sports facilities were distinguished, and relying on the international literature [26], different weighting values were assigned to them (Table 1). The availability of sports facilities in the settlements were studied by applying the complex indicator obtained in this way, using absolute as well as relative values based on population number with special attention to the differences between settlements with regard to size and legal status.

## 3. Results

In terms of the availability of sports facilities in settlements of different sizes, the strong influence of the size of the settlement is clearly visible. The indicator reflecting availability in a complex way (Table 2) is lower as the population decreases, and this process can be clearly observed in all three counties (greater differences between the counties are hardly noticeable).

Data on sports facilities in different size categories clearly reflect the strong determining role of the settlement size (Table 3). In the case of sports facilities in schools, larger gyms are concentrated primarily in schools located in settlements with more than 10,000 inhabitants, while in smaller settlements, facilities of less than 450 square metres in size play a decisive role. In the case of settlements with fewer than 1000 inhabitants, there are primary schools in only eight of the 19 settlements, and 7 of them also have gyms (although these are of the smallest size).

Similar trends can be also observed in case of sports facilities serving the purposes of the general population (Table 4). There are almost no such facilities in smaller settlements (unfortunately these include settlements with populations between 2000 and 5000), and the full range of facilities (at least one tennis court, a swimming pool, and a fitness room) is typical only for settlements with more than 10,000 inhabitants.

In case of facilities used for competitive sport (Table 5), a slightly more positive picture can be found. On the one hand, it is true that the artificial grass pitches, as well as the sports halls that ensure the cultivation of gym sports at a high level, are typical only in settlements with populations over 10,000 people. A total of 44.4% of settlements with populations between 5000 and 10,000 have sports halls, and 11.1% have large-sized artificial turf pitches, while the lack of natural grass pitches was observed only in cases of settlements with fewer than 1000 inhabitants.

Considering the inequality of the distribution of the three large types of sports facilities (Table 6), the greatest difference (and thus the most unequal distribution) was observed in the case of recreational sport, followed by school sport, and competitive sport. 

The next major unit of the research was devoted to analysing the impact of the administrative role of municipalities. Only three categories based on population size were included in this comparison, as it was only in these categories that we can find both district seats and non-district seat settlements. In terms of the complex indicator reflecting the level of availability (Table 7), the strong impact of the administrative role can be demonstrated: the values of district seats in all three categories of population were found to be significantly higher than the data for all settlements.

The main role of district seats is also shown in the quality indicators of individual sports facilities. Firstly, schools in district centres have larger gyms than those in other settlements (Table 8) and secondly, in the case of recreational sports facilities, the better situation of district seats can be clearly seen (Table 9).

Thirdly, quality differences can also be observed in terms of competitive sports (it was not possible to do calculations here in most cases because of the low number of elements), although to the smallest extent (Table 10). 

## 4. Discussion

Revealing the significant influencing impact of the population size may be regarded as one of the most important findings of the section on results (larger settlements—more facilities) which has been also highlighted by previous international research. This, however, can be traced back to different causes depending on the type of the sports facilities. In the case of school gyms (Table 3), the primary reason is to be found in the number of enrolled students of the educational institutions: in the settlements with a lower population number the schools operate with maximum 100–200 students, and in these cases building a facility with a larger floor area is not efficient both in terms of utilisation and maintenance. Furthermore, in the settlements with higher population numbers, these facilities are often also used by a wide range of sports clubs (e.g., basketball training/matches), which in fact made it necessary to design a playing field with a size that meets the requirements of specific sports.

In the case of the sports facilities which are designed to meet the needs of the wider public (Table 4), this phenomenon can be primarily explained by the fact that the population living in larger settlements has a higher income in general, and thus they are more likely to be ready to pay for using these facilities. The similar data observed in the case of competitive sports (Table 5) is due to the fact that sports associations which require this kind of infrastructure (providing training opportunities or organising matches) for participation in various championships are mostly operated in larger settlements. Looking at the data for the settlements with lower population numbers, it may be established that they lag behind the least in the case of large football pitches with natural grass cover (e.g., almost every settlement has such a facility). This can be explained by the fact that football, as the most popular as well as perhaps the cheapest sport to play, and also as an important factor in social cohesion, is present in almost every settlement (e.g., there are football clubs), and this makes it necessary to create this type of facility.

The main cause of the inequalities observed in the distribution of the three major sports events (Table 6) is that visiting recreational sports facilities requires the most considerable financial resources, whereas, as pointed out before, football pitches, as one of the foundations of competitive sport, are present in almost all municipalities. This statement is confirmed by the fact that the highest concentration of the three large-sized facility types for recreational sports can be observed in the case of tennis infrastructure, which requires the most financial resources.

The findings related to the study of the administrative role of the municipalities (e.g., whether the given settlement is a district centre) can be justified by several factors. In the case of school gyms (Table 8), the most important explanation is that, due to the central administrative role of such settlements, these schools in most cases have a higher number of students (many of them are commuting there from surrounding settlements), and of course this has made it necessary to build larger gyms.

Regarding recreational sports facilities (Table 9), however, it is a very interesting fact that in cases of settlements with populations between 5000 and 10,000, the differences are much greater than in cases of settlements with 10,000–50,000 people, which is probably due to the fact that the district seat is much more important in cases of the former category (e.g., with many more additional functions) than in cases of settlements with 10,000–50,000 people.

The third area related to the importance of the administrative role is constituted by competitive sports, where the impact of district seats is observable but only moderate (Table 10), which can be essentially explained by two facts. For the reasons mentioned in the explanation to the previous table, the values of district seats in the largest category of settlements hardly exceeded the values for all settlements combined, and in the case of natural grass pitches, there was no significant difference found between the data of all settlements and district seats, due to the explanation given in the paragraph relating to the number of population of settlements.

## 5. Conclusions

The most important findings of the study could be summarised as follows. Firstly, the spatial distribution of sports facilities is significantly influenced by the size of the settlements: larger settlements have more diverse and higher-quality sports infrastructure (e.g., larger gyms, more fitness centres). In our opinion this can be attributed to the increased demand from the population and the associated higher purchasing power. Secondly, the smallest difference between settlements of different sizes can be observed in the case of competitive sports which can be explained by football pitches being available in almost all settlements (which can be explained by the relative cheapness and general popularity of football). Thirdly, the administrative status of settlements can also be considered as an important factor: settlements that serve as district seats were found to have better facilities, which is due to the important role of these settlements within their districts.

## Figures and Tables

**Table 1:** Multiplication indexes used for the calculation of the complex indicators.

Sports Facilities: Type	Sports Facilities: Characteristics	Weighting Value
	smaller than 450 m^2^	1.0
school gym	450–900 m^2^	1.6
	larger than 900 m^2^	2.2
artificial turf	outdoors	1.6
small pitch	indoors	2.2
tennis court	outdoors	1.6
	indoors	2.0
swimming pool	25 to 33 m, outdoor	1.6
	25 to 33 m, indoor	2.0
	50 m, outdoor	1.8
	50 m, outdoor	2.4
fitness room		2.4
sports hall		3.0
football	natural grass	1.8
large pitch	artificial turf	2.2

Source: own work.

**Table 2:** The settlement average of the value of the complex indicator reflecting the availability of sport facilities for each settlement in different size categories in each county of the Észak-Alföld region.

Number of Inhabitants	Hajdú-Bihar County	Jász-Nagykun-Szolnok County	Szabolcs-Szatmár-Bereg County	Northern Great Plain Region
less than 1000 inhabitants	0.9	2.4	1.8	1.7
1000–2000 inhabitants	2.8	3.6	3.3	3.3
2000–5000 inhabitants	4.3	5.3	6.4	5.4
5000–10,000 inhabitants	14.0	12.2	13.8	13.5
10,000–50,000 inhabitants	28.2	29.9	31.9	29.9
more than 50,000 inhabitants	230.7	101.6	141.2	157.8

Source: own calculation based on method in Table 1.

**Table 3:** The size of existing gyms in schools located in different categories of settlements in the Northern Great Plain region (%).

Number of Inhabitants	Smaller Than 450 m^2^	450–900 m^2^	Larger Than 900 m^2^
less than 1000 inhabitants	100.0	0.0	0.0
1000–2000 inhabitants	86.7	13.3	0.0
2000–5000 inhabitants	78.6	21.4	0.0
5000–10,000 inhabitants	60.4	33.3	6.3
10,000–50,000 inhabitants	67.3	22.4	10.3
more than 50,000 inhabitants	65.0	20.0	15.0

Source: own collection.

**Table 4:** The absolute number and average of sports facilities serving the purposes of the population on settlements of different sizes in Northern Great Plain region (average—value per settlement).

		A	B	C	D	E	F
outdoor	absolute number (pcs)	0	0	2	14	39	47
tennis courts	number per settlement	0.0	0.0	0.1	0.8	2.2	15.7
indoor	absolute number (pcs)	0	0	1	0	5	18
tennis courts	number per settlement	0.0	0.0	0.0	0.0	0.3	6.0
fitness	absolute number (pcs)	0	1	1	11	36	35
rooms	number per settlement	0.0	0.0	0.0	0.6	2.0	11.7
	25 to 33 m, outdoor	0	0	0	6	10	0
swimming	25 to 33 m, indoor	0	0	4	3	8	7
pools (pcs)	50 m, indoor	0	0	0	1	6	3
	50 m, indoor	0	0	0	1	3	3

A—less than 1000 inhabitants; B—1000–2000 inhabitants; C—2000–5000 inhabitants; D—5000–10,000 inhabitants; E—10,000–50,000 inhabitants; F—more than 50,000 inhabitants. Source: own collection.

**Table 5:** The absolute number and average of sports facilities used for competitive sport in settlements of different sizes in Northern Great Plain region.

		A	B	C	D	E	F
sport	absolute number (pcs)	0	0	1	8	23	7
halls	number per settlements	0.0	0.0	0.0	0.4	1.3	2.3
football large pitch	absolute number (pcs)	12	16	27	29	34	24
natural grass	number per settlements	0.6	1.0	1.2	1.6	1.9	8.0
football large pitch	absolute number (pcs)	0	0	0	2	9	7
artificial turf	number per settlements	0.0	0.0	0.0	0.1	0.5	2.3

A—less than 1000 inhabitants; B—1000–2000 inhabitants; C—2000–5000 inhabitants; D—5000–10,000 inhabitants; E—10,000–50,000 inhabitants; F—more than 50,000 inhabitants. Source: own collection.

**Table 6:** The percentage of the average points given for the availability of sport facilities in municipalities in each category of population, within the given type of facility (%).

Number of Inhabitants	School Sports	Recreational Sports	Competitive Sports	All of the Facilities
less than 1000 inhabitants	0.53	0.11	2.47	0.78
1000–2000 inhabitants	1.76	0.22	4.04	1.57
2000–5000 inhabitants	2.46	0.78	5.17	2.30
5000–10,000 inhabitants	5.98	4.21	10.11	6.11
10,000–50,000 inhabitants	13.18	12.86	18.65	14.30
more than 50,000 inhabitants	76.10	81.82	59.55	74.95
total	100.00	100.00	100.00	100.00

Source: own collection.

**Table 7:** The average points given for the availability of sports facilities in district seats in comparison with all settlements in the same size category.

Number of Inhabitants	All of the Settlements	Districts Seats
2000–5000 inhabitants	5.4	10.5
5000–10,000 inhabitants	13.5	18.3
10,000–50,000 inhabitants	29.9	31.4

Source: own calculation based on method in Table 1.

**Table 8:** The distribution of gyms in all settlements and in district seats in the same population category according to the size of the gyms (%).

		Smaller Than 450 m2	450–900 m^2^	Larger Than 900 m^2^
	2000–5000 inhabitants	78.6	21.4	0.0
all of the settlements	5000–10,000 inhabitants	60.4	33.3	6.3
	10,000–50,000 inhabitants	67.3	22.4	10.3
	2000–5000 inhabitants	71.4	28.6	0.0
districts seats	5000–10,000 inhabitants	57.1	35.7	7.2
	10,000–50,000 inhabitants	64.8	23.1	12.1

Source: own collection.

**Table 9:** The distribution of recreational sports facilities in all settlements and in district seats in the same population category.

		All Settlements		District Seats	
		A	B	A	B
outdoor	absolute number (pcs)	14	39	10	36
tennis courts	number per settlement	0.8	2.2	1.2	2.4
fitness	absolute number (pcs)	11	36	8	33
rooms	number per settlement	0.6	2.0	1.0	2.2
	25–33 m (pcs)	9	18	7	16
swimming pools	25–33 m (number per settlement)	0.5	1.0	0.9	1.1
	50 m (pcs)	x	9	x	8
	50 m (number per settlement)	x	0.5	x	0.5

A—5000–10,000 inhabitants; B—10,000–50,000 inhabitants; x—due to the low number of items it cannot be counted. Source: own collection.

**Table 10:** The distribution of competitive sports facilities for all settlements and in district seats in the same population category.

		All of the Settlements			District Seats		
		A	B	C	A	B	C
sports halls	absolute number (pcs)	x	8	23	x	6	19
	number per settlement	x	0.4	1.3	x	0.8	1.3
football large pitch,	absolute number (pcs)	27	29	34	4	14	29
natural grass	number per settlement	1.2	1.6	1.9	1.3	1.7	1.9
football large pitch,	absolute number (pcs)	x	x	9	x	x	9
artificial turf	number per settlement	x	x	0.5	x	x	0.6

A—2000–5000 inhabitants; B—5000–10,000 inhabitants; C—10,000–50,000 inhabitants; x—due to the low number of items it cannot be counted. Source: own collection.

## Data Availability

Not applicable.
